# Primary clear cell adenocarcinoma of female urinary tract: a case report and literature review

**DOI:** 10.1186/s12905-022-01835-6

**Published:** 2022-06-24

**Authors:** Jianguang Miao, Jiebin Hu, Jilin Wu, Wei Guo, Jinbo Chen, Jin Li

**Affiliations:** 1 Department of Urology, Xiangtan Central Hospital, Xiangtan, 411100 Hunan China; 2 Department of Urology, Xiangya Hospital, Central South University, Changsha, 410000 Hunan China

**Keywords:** Case report, Clear cell adenocarcinoma, Urethra

## Abstract

**Background:**

Primary clear cell adenocarcinoma of the urethra is extremely rare, reported only in single case reports, and its histological origin is not clear. There is no standard treatment for CCAU at present, and surgery is still the main treatment for CCAU without distant metastasis.

**Case presentation:**

A 67-year-old female complained of gross hematuria with frequent micturition and urgency. No urethral diverticulum was found by cystoscopy or MRI, and the mass grew around the urethra. Urethral and anterior pelvic viscera resection was performed. Clear cell adenocarcinoma was confirmed by immunohistochemistry after the operation, and no recurrence or metastasis was found after one year of follow-up.

**Conclusion:**

CCAU is very rare, and most cases originate from the urethral diverticulum and some may also originate from tissues around the urethra. For CCAU patients without distant metastasis, the main treatment is still surgery, and radiotherapy and chemotherapy can be performed for patients with distant metastasis. Gene detection may provide guidance for the precise chemotherapy of CCAU.

## Introduction

Primary clear cell adenocarcinoma of the urethra (CCAU) is extremely rare and has been reported in only single case reports. Most reported CCAU cases are highly correlated with urethral diverticula. Cystoscopy and MRI can be used to identify urethral diverticula and masses in diverticula. We report a new female CCAU patient. No urethral diverticulum was found by cystoscopy or MRI, and the mass grew around the urethra. The whole urethra could be clearly displayed by MRI.

## Case presentation

A 67-year-old female complained of gross hematuria with frequent micturition and urgency. No abnormalities were found during cystoscopy at the outpatient department of Xiangtan Central Hospital. Because the patient refused to undergo ultrasound, CT and other related examinations, she was diagnosed with urinary infection and was given antibiotics and other related treatment. The symptoms did not improve, and she was hospitalized.

**Fig. 1:**
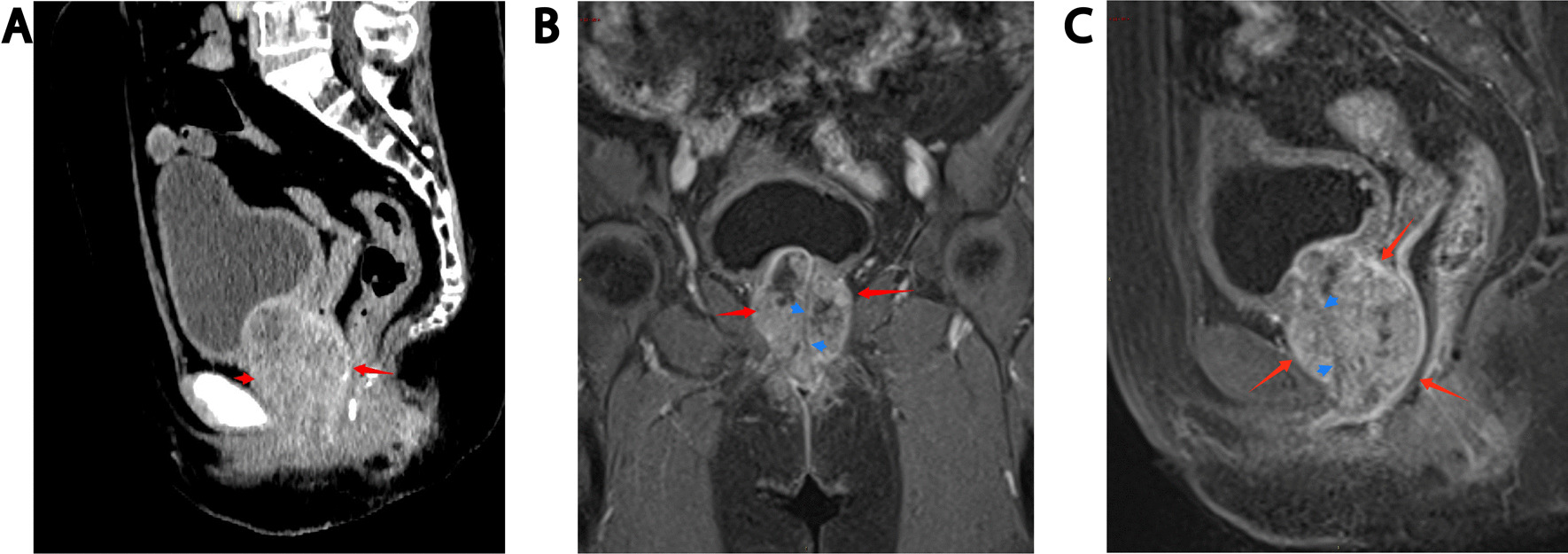
CT showed a soft tissue mass (red arrow) with a size of approximately 60*47*53 mm below the bladder and in front of the vagina (**A**), MRI showed a round mass (red arrrow) around the urethra with a size of approximately 45*49*62 mm, with a capsule and clear boundary, and a urethral shadow (blue arrow) could be seen in the center of the lesion (**B**, **C**)

**Fig. 2:**
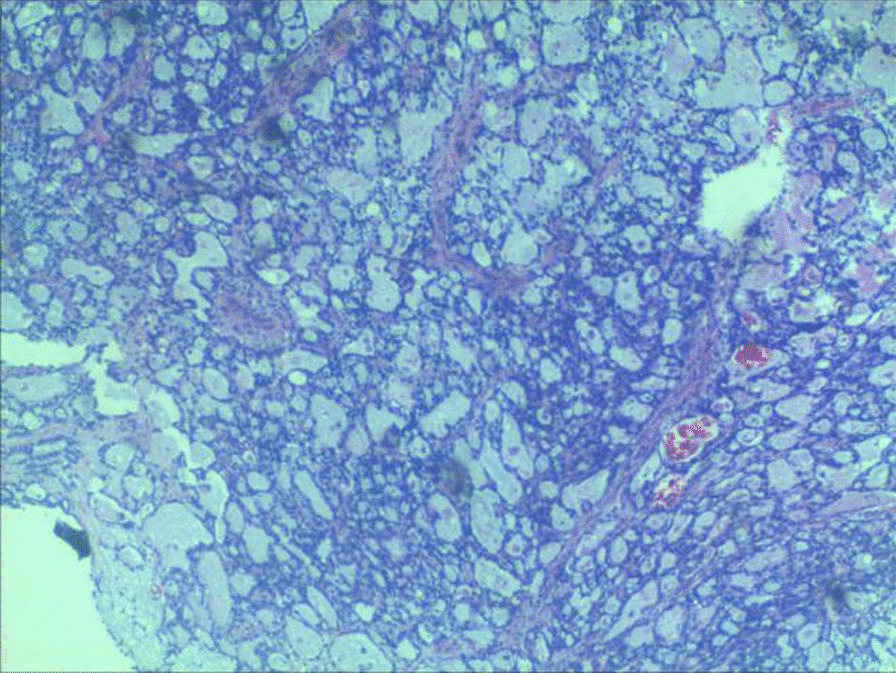
HE staining (100 ×) showed tumor cells had vacuolated, clear cytoplasm and basally located pleomorphic nuclei without prominent nucleoli, numerous tumor cells with clear cytoplasm, a key feature of CCAU

**Fig. 3:**
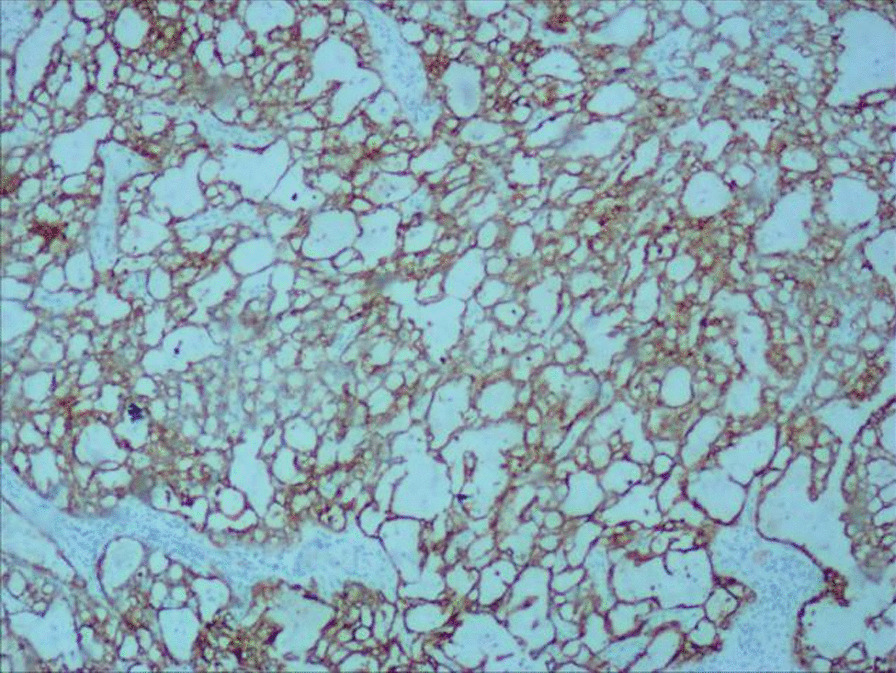
IHC (100 ×) demonstrated that tumor cells diffusely and strongly expressed CK7 in a membranous pattern

After admission, CT showed a soft tissue mass with a size of approximately 60*47*53 mm below the bladder and in front of the vagina (see Fig. 1A), MRI showed a round mass around the urethra with a size of approximately 45*49*62 mm, with a capsule and clear boundary, and a urethral shadow could be seen in the center of the lesion (see Fig. 1B, C). No urethral diverticulum or mass was found under cystoscopy, and obstructive changes such as trabecula were found in the bladder. Before the operation, the patient underwent transperineal mass biopsy guided by ultrasound. Pathology and immunohistochemistry showed clear cell adenocarcinoma. After being transferred to Xiangya Hospital of Central South University for PET-CT, no lymph nodes or distant metastases were found. The patient underwent urethral resection combined with anterior pelvic organ resection and ileal conduit construction. The excised specimen showed a white carrion-like mass around the urethra with an obvious capsule. She was diagnosed as clear cell adenocarcinoma of the urethra involving the vaginal wall. HE staining showed tumor cells had vacuolated, clear cytoplasm and basally located pleomorphic nuclei without prominent nucleoli, numerous tumor cells with clear cytoplasm (Fig. 2, × 100, Olympus BX43, microscope, objective HI Plan × 10). IHC demonstrated that tumor cells diffusely and strongly expressed CK7 in a membranous pattern (Fig. 3, × 100, Olympus BX43, microscope, objective HI Plan × 10) and negative for GATA3, GK20, P63, CDX-2, PAX-2, CA9. The patient underwent genetic testing and was provided a drug regimen for chemotherapy (see Table 1). The gene ARID1A p.l1160x (ex13) was detected in the tumor of the patient, suggesting that the patient may benefit from everolimus and bevacizumab, and the patient had LOH (HLA-A * 02:01 deletion), suggesting that the patient is less likely to benefit from immune checkpoint inhibitor treatment. She refused further chemotherapy or targeted therapy. One year after the operation, no recurrence was found.

**Table 1:** suggestions for chemotherapy drugs suggested by gene detection; Results were obtained from the SanWay Clinical Laboratory, Changsha, Hunan, China

Recommended drugs	Cyclophosphamide, tegafur, capecitabine, thioguanine, azathioprine, mercaptopurine, taxol, docetaxel, cytarabine
Optional drugs	Cisplatin, lobaplatin, carboplatin, methotrexate, cetippine, gemcitabine, SN-38, irinotecan, fluorouracil, etoposide, cetuximab
Use drugs with caution	Doxorubicin, epirubicin, daunorubicin, idarubicin, pirarubicin, capecitabine + calcium folinate, fluorouracil + calcium folinate, tegafur + calcium folinate

## Discussion and conclusions

Clear cell adenocarcinoma of the female urethra is very rare, and its tissue origin is not clear. It may originate from urothelial metaplasia, Müllerian rests or periurethral glands [1], and it is highly correlated with urethral diverticulum [2–5]. Olivia [3] performed a clinicopathological analysis of 19 patients with CCAU and found that 12 of the cases originated from urethral diverticulum, which shows that CCAU is highly correlated with urethral diverticulum. The reason may be urothelial metaplasia [4] caused by repeated chronic inflammatory stimulation caused by urethral diverticulum. However, there were also some cases in which urethral tumor and diverticulum [6] were not found during cystoscopy and imaging examination in patients diagnosed with CCAU, suggesting that the tumor grew along the periphery of urethra and may have originated from glands around urethra. In our report, cystoscopy, MRI and CT showed no urethral mass or diverticulum, which also suggested that the CCAU might have originated from the glands around the urethra.

The main symptom of CCAU is hematuria, and other symptoms include waiting for urination, dysuria and repeated urinary infection [7, 8]. Because of the diversity and nonspecificity of symptoms, the preoperative diagnosis of CCAU is difficult. Imaging examinations such as CT and MRI can provide guidance for its diagnosis and surgical methods [5, 9]. Kim [5] reported distinctive MRI findings that allowed the differentiation of CCAU from NACU (nonadenocarcinoma of the urethra): 1. There is a high correlation between the CCAU and urethral diverticulum. 2. CCAU has a lower height-to-width ratio (0.74–1.09). 3. There is a larger proportion of preserved urethra. However, due to the small number of cases, more studies are needed to confirm these findings. Our MRI results show that the height-to-width ratio is higher than that in Kim's research (1.27), and the whole urethra can be retained in the tumor, but no urethral diverticulum was found. Cystoscopy is a routine method for obtaining a definite diagnosis before surgery. It can find the mass in the urethra and take a biopsy to make a definite diagnosis. However, there are also CCAU patients whose cystoscopy is normal [6], so they need to undergo biopsy of the mass under the guidance of ultrasound. Nevertheless, the final diagnosis of CCAU relies on histological and immunohistochemical analyses. CCAU has characteristic microscopic features, including enlarged tumor cells containing abundant clear cytoplasm with conspicuous vacuoles, hobnail patterned cells, and hyaline globules.

The 5-year survival rate of CCAU is low due to its high invasiveness [10]. The main treatment for patients without distant metastasis is surgery, including 1. urethral resection; 2. urethral resection combined with anterior pelvic organ resection; and 3. urethral resection combined with anterior pelvic organ resection and pelvic lymph node dissection. However, in some reports [6, 10, 11], anterior pelvic organ resection combined with pelvic lymph node dissection was performed. Patel [12] studied the prognostic factors of CCAU patients, suggesting that surgery can improve the OS and DSS of patients, but there is no standard surgical protocol for CCAU. We performed urethral resection combined with anterior pelvic organ resection for this patient because no lymph node involvement was found by PET-CT. The patient had no recurrence after 1 year of follow-up. Urethrectomy resection in combination with anterior exenteration may be considered a good option for thetreatment for CCAU [10].

Some studies [10, 13] suggest that pelvic consolidation radiotherapy can be performed for patients with positive lymph nodes, while chemotherapy is rarely used. Gogus [14] and others gave a postoperative patient three cycles of methotrexate, vincristine, epirubicin and cisplatin (MVEC) chemotherapy. This patient had confirmed disease progression and died 10 months after the operation. Liu [15] and others treated a metastatic CCAU patient with palliative chemotherapy of cisplatin and gemcitabine and achieved disease control for 2 years. However, reports on chemotherapy for clear cell carcinoma of the urethra are limited, and there is no unified chemotherapy plan at present. Nevertheless, with the development of tumor gene detection technology, drugs sensitive to tumor cells can be selected for precise chemotherapy. Shields [16] reported that a patient with metastatic CCAU was treated with paclitaxel/bevacizumab chemotherapy for 11 cycles according to the results of gene detection and achieved 12 months of progression-free survival. Lymphedema of the lower extremities was the sole complication. It was suggested that tumor gene detection may provide a new direction and reference for the precise chemotherapy of CCAU.

CCAU is very rare, and most cases originate from the urethral diverticulum and some may also originate from tissues around the urethra. For CCAU patients without distant metastasis, the main treatment method is still surgery, and radiotherapy and chemotherapy can be performed for patients with distant metastasis. However, reports on chemotherapy for clear cell carcinoma of the urethra are limited, and there is no standard chemotherapy plan. Gene detection may provide guidance for the precise chemotherapy of CCAU.

## Data Availability

The datasets used and analysed during the current study are available from the corresponding author on reasonable request.

## Abbreviations

CCAU: Clear cell Adenocarcinoma of the urethra
MRI: Magnetic resonance examination
NACU: Nonadenocarcinoma of the Urethra
